# Tröger’s Base-Derived Thermally Activated Delayed Fluorescence Dopant for Efficient Deep-Blue Organic Light-Emitting Diodes

**DOI:** 10.3390/molecules28124832

**Published:** 2023-06-17

**Authors:** Ze-Ling Wu, Xin Lv, Ling-Yi Meng, Xu-Lin Chen, Can-Zhong Lu

**Affiliations:** 1 State Key Laboratory of Structural Chemistry, Fujian Institute of Research on the Structure of Matter, Chinese Academy of Sciences, Fuzhou 350002, China; wuzeling20@mails.ucas.ac.cn (Z.-L.W.); xmlvxin@fjirsm.ac.cn (X.L.); lymeng@fjirsm.ac.cn (L.-Y.M.); 2 Fujian Science & Technology Innovation Laboratory for Optoelectronic Information of China, Fuzhou 350108, China; 3 Xiamen Key Laboratory of Rare Earth Photoelectric Functional Materials, Xiamen Institute of Rare Earth Materials, Haixi Institutes, Chinese Academy of Sciences, Xiamen 361021, China; 4 University of Chinese Academy of Sciences, Beijing 100049, China

**Keywords:** deep-blue emission, Tröger’s base, thermally activated delayed fluorescence, spin-orbit coupling constant, organic light-emitting diode

## Abstract

The development of efficient deep-blue emitters with thermally activated delayed fluorescence (TADF) properties is a highly significant but challenging task in the field of organic light-emitting diode (OLED) applications. Herein, we report the design and synthesis of two new 4,10-dimethyl-6H,12H-5,11-methanodibenzo[*b,f*][1,5]diazocine (**TB**)-derived TADF emitters, **TB-BP-DMAC** and **TB-DMAC**, which feature distinct benzophenone (**BP**)-derived acceptors but share the same dimethylacridin (**DMAC**) donors. Our comparative study reveals that the amide acceptor in **TB-DMAC** exhibits a significantly weaker electron-withdrawing ability in comparison to that of the typical benzophenone acceptor employed in **TB-BP-DMAC**. This disparity not only causes a noticeable blue shift in the emission from green to deep blue but also enhances the emission efficiency and the reverse intersystem crossing (RISC) process. As a result, **TB-DMAC** emits efficient deep-blue delay fluorescence with a photoluminescence quantum yield (PLQY) of 50.4% and a short lifetime of 2.28 μs in doped film. The doped and non-doped OLEDs based on **TB-DMAC** display efficient deep-blue electroluminescence with spectral peaks at 449 and 453 nm and maximum external quantum efficiencies (EQEs) of 6.1% and 5.7%, respectively. These findings indicate that substituted amide acceptors are a viable option for the design of high-performance deep-blue TADF materials.

## 1. Introduction

Over the past few decades, organic light-emitting diodes (OLEDs) have attracted extensive academic and industrial attention in solid-state lighting and flexible ultra-thin displays due to their intrinsic merits of being ultra thin and lightweight as well as having high brightness and flexibility with infinite design possibilities [1,2,3]. Electroluminescence (EL) materials which determine the main device performance, such as emission color, EL efficiency, and operating lifetime, are the core part of OLEDs [4,5,6]. Thermally activated delayed fluorescent (TADF) materials, which can harvest triplet (T_1_) and singlet (S_1_) excitons for light emission via efficient reverse intersystem crossing (RISC), have emerged as promising fluorescent dyes for the construction of efficient OLEDs [7]. The thermally activatable RISC process requires a small amount of singlet–triplet energy splitting (∆E_ST_), which can be achieved in donor–acceptor (D–A) molecules in which the highest occupied molecular orbital (HOMO) and the lowest unoccupied molecular orbital (LUMO) distributions are well separated [8,9,10,11,12,13].

The development of deep-blue emitters is crucial in realizing full-color OLED displays with a high performance [14,15,16,17,18,19,20,21,22]. When designing deep-blue TADF emitters with D–A features, a weak CT character and a confined conjugation length are necessary to restrict the emission color to the deep-blue region [23]. However, the weakened CT character generally leads to a relatively large ΔE_ST_ and a small rate constant for reverse intersystem crossing (k_RISC_), which would negatively affect device efficiency and stability in OLEDs [24]. Hence, it is challenging, and strict design principles must be followed to balance the trade-offs between ΔE_ST_, the RISC rate, the photoluminescence quantum yield (PLQY), and color purity of deep-blue TADF emitters [25,26,27].

Benzophenone-derived units are well known for their ability to undergo efficient intersystem crossing (ISC) due to their strong spin–orbit coupling [28]. This property makes them a highly attractive option as acceptor blocks for constructing TADF emitters. Although many benzophenone-based TADF materials have been reported to emit green to red light, achieving blue TADF materials has been challenging due to their strong electron-withdrawing ability [29,30,31,32,33,34,35,36]. Recently, Tang et al. modified the para-position of the carbonyl group of benzophenone with N to obtain sky blue TADF materials with satisfactory performances [37,38,39]. Duan and Adachi et al. proposed that minimizing the distance between heteroatoms in donors and acceptors can significantly enhance the contribution of heteroatoms to the natural orbitals of singlet and triplet states. This, in turn, can promote the proportion of the n-π* transition and increase the SOC between them, resulting in an improved RISC rate [40].

Tröger’s base (**TB**) is a C_2_-symmetric, unique, V-shaped twisted scaffold [41] that has been utilized to create amorphous host materials with a high thermal stability for OLEDs [42]. In this work, we designed and synthesized two novel 4,10-dimethyl-6H,12H-5,11-methanodibenzo[*b,f*][1,5]diazocine (**TB**)-derived TADF molecules with symmetrical D–A features, namely **TB-BP-DMAC** and **TB-DMAC**, bearing different benzophenone-derived acceptors and the same dimethylacridin (**DMAC**) donors. Compared to the typical benzophenone acceptor used in **TB-BP-DMAC**, the amide acceptor in **TB-DMAC** has a much weaker electron-withdrawing ability, resulting in a noticeable blue shift in the emission from green (with a spectrum peak at 517 nm for **TB-BP-DMAC**) to deep blue (with a spectrum peak at 467 nm for **TB-DMAC**). Additionally, **TB-DMAC** exhibits a higher photoluminescence quantum yield (PLQY) of 50.4% and a faster RISC rate of 6.13 × 10^5^ s^−1^ compared to those of **TB-BP-DMAC** (PLQY = 31.5%, k_RISC_ = 5.83 × 10^5^ s^−1^). In a doped OLED employing **TB-DMAC** as the emitter, the deep-blue emission had a maximum external quantum efficiency (EQE) of 6.1%, which is higher than the 3.9% EQE of the **TB-BP-DMAC**-based green-emitting device.

## 2. Results and Discussion

### 2.1. Synthesis and Characterization

**Scheme 1:**
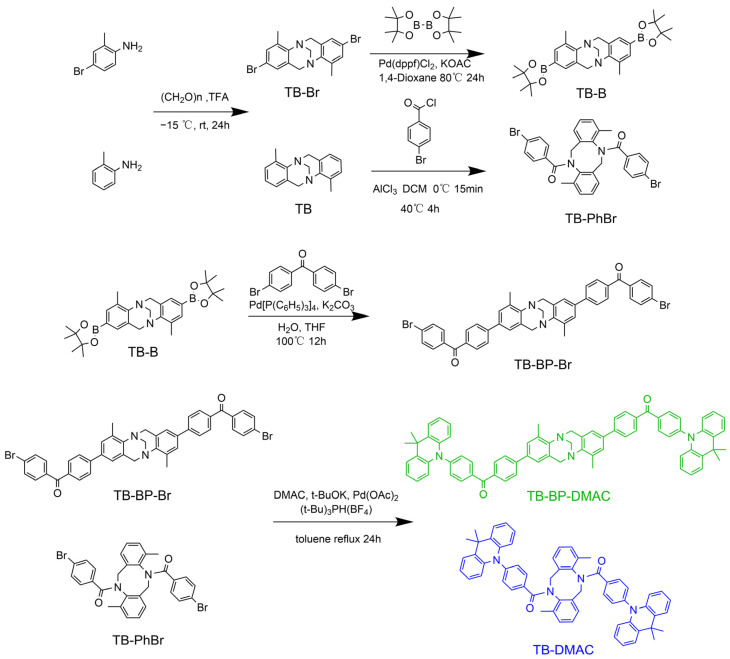
Synthetic route of **TB-BP-DMAC** and **TB-DMAC**.

Synthetic routes and detailed synthetic procedures of the target compounds are described in Scheme 1 and the Section 3, respectively. The intermediate **TB-BP-Br** was synthesized in three steps, including condensation, Miyaura borylation reaction, and Suzuki coupling. Meanwhile, the intermediate **TB-PhBr** was obtained via two steps, namely condensation and N-acylation. The Pd-catalyzed Buchwald–Hartwig cross-coupling of the compounds **TB-PhBr** and **TB-BP-Br** with 9,9-dimethyl-9,10-dihydroacridine (**DMAC**) resulted in good yields of **TB-BP-DMAC** and **TB-DMAC**, respectively. All the newly synthesized materials were purified by column chromatography. These compounds are quite air stable in both the solid state and in solution. The chemical structures of the newly synthesized compounds were characterized using ^1^H NMR spectra, as shown in Supporting Information. The target compounds **TB-BP-DMAC** and **TB-DMAC** were further purified by vacuum sublimation before characterization and device fabrication were performed. The thermal stability of these compounds was examined by a thermal gravimetric analysis (TGA) under a nitrogen atmosphere. As depicted in Figure S7, both **TB-BP-DMAC** and **TB-DMAC** exhibit a high thermal stability, with decomposition temperatures (Td, corresponding to a 5% weight loss) of 270 and 299 °C, respectively. These values are sufficiently high to enable film and device fabrication through high-vacuum thermal deposition.

### 2.2. Theoretical Investigation

**Figure 1:**
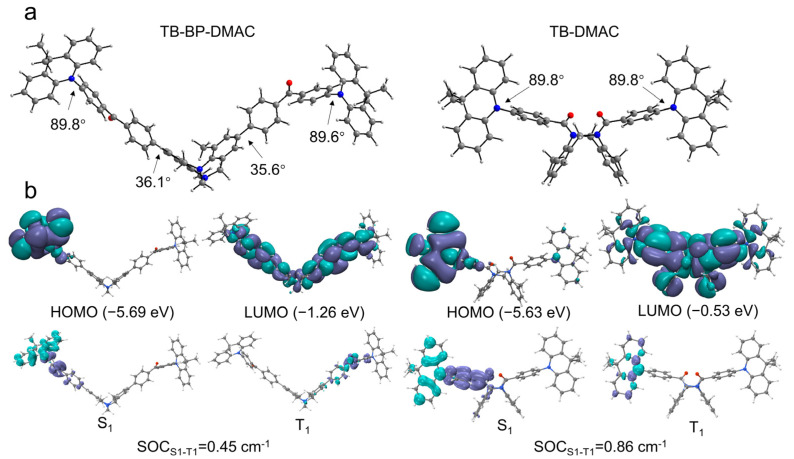
(**a**) The structure and torsion angle of **TB-BP-DMAC** and **TB-DMAC** in ground state, respectively; (**b**) calculated HOMO, LUMO, S_1_ and T_1_ distributions, and SOC constants of **TB-BP-DMAC** and **TB-DMAC**.

The geometric structures and electronic properties of these molecules have been investigated by DFT and time-dependent DFT (TD-DFT) calculations at the BMK/6-31G** level. The optimized ground-state molecular geometries revealed symmetrical twisted structures and nearly perpendicular donor–acceptor (D–A) conformations of these two molecules, with dihedral angles of 89.6–89.8° between the **DMAC** units and the bridging phenyl rings, respectively (Figure 1a). Such large D–A torsion angles typically promote effective spatial separation between the frontier molecular orbitals, thereby facilitating a small ΔE_ST_, which is crucial for the reverse intersystem crossing (RISC) process. The highest occupied molecular orbitals (HOMOs) of these compounds are primarily confined at electron-donating **DMAC** moieties, while the LUMOs are mainly distributed over the electron-deficient benzophenone moiety, implying a good separation of HOMO and LUMO distributions (Figure 1b). The TD-DFT calculations revealed that the S_1_ states of **TB-BP-DMAC** and **TB-DMAC** have predominant CT characteristics, while their T_1_ states are both locally excited states (^3^LE). Compared with that of **TB-DMAC**, the molecular conjugation length of **TB-BP-DMAC** is significantly extended by adding a bridging phenyl ring and adjusting the connection position, resulting in a much deeper LUMO level than that of **TB-DMAC** (−1.26 versus −0.53 eV). Based on the TD-DFT calculations, the SOC constants for S_1_ and T_1_ states were found to be 0.45 and 0.86 cm^−1^ for **TB-BP-DMAC** and **TB-DMAC**, respectively. These significant SOC interactions may be attributed to the different excitation natures of these lowest excited states, which could play a crucial role in the RISC processes.

### 2.3. Electrochemical Properties

The electrochemical properties of these compounds were characterized by cyclic voltammetry (CV) (Figure S8). From the onsets of the oxidation curves, the HOMO energy levels of **TB-BP-DMAC** and **TB-DMAC** were calculated to be −5.26 and −5.28 eV, respectively. The close HOMO levels of two compounds can be ascribed to the use of identical donors. The LUMO energy levels were then determined to be −2.40 and −2.08 eV based on the HOMO levels and the optical band gaps (2.86 and 3.19 eV, vide infra) which were evaluated from the onsets of the absorption spectra. The LUMO energy level of **TB-BP-DMAC** was much deeper than that of **TB-DMAC** due to the significantly extended π-conjugation, which is consistent with the results of the quantum chemical calculations.

### 2.4. Photophysical Properties

**Figure 2:**
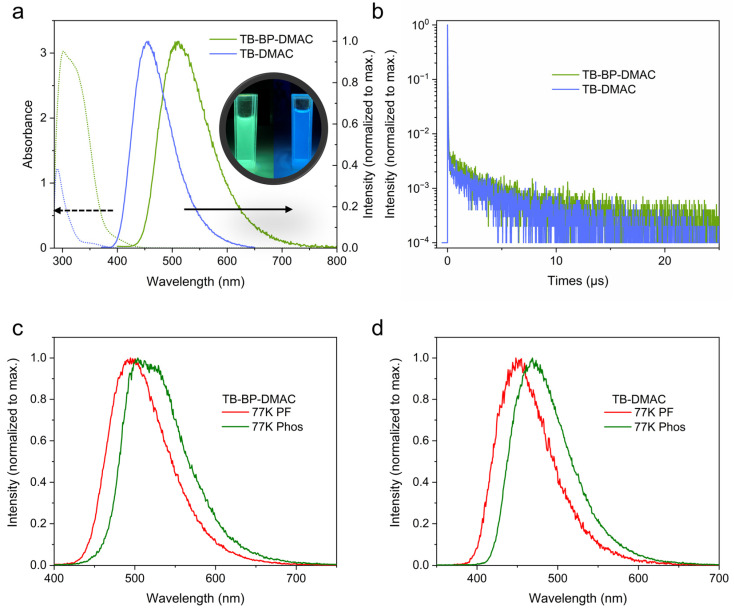
(**a**) Absorption and PL spectra (λ_ex_ = 300 nm) of **TB-BP-DMAC** and **TB-DMAC** in toluene (c = 1 × 10^−5^ M) measured at room temperature (a dashed arrow and a solid arrow indicate the absorption and PL spectra, respectively). Inset: the photographs of **TB-BP-DMAC** (**left**) and **TB-DMAC** (**right**) in toluene (1 × 10^−5^ M) under excitation of 365 nm UV light, taken with an iPhone; (**b**) transient PL decay curves in 20 wt%-doped PPF films at room temperature; (**c**,**d**) time-resolved PL spectra in 20 wt%-doped PPF films at 77 K (PF: prompt fluorescence; Phos: phosphorescence).

The photophysical properties of these emitters were investigated in toluene (c = 10^−5^ M). Figure 2a displays the absorption spectra of **TB-BP-DMAC** and **TB-DMAC** in toluene. The intense higher-energy absorption bands observed at wavelengths below 380 nm and 335 nm, respectively, are primarily attributed to the spin-allowed π-π* transitions. Additionally, these molecules exhibit weak and broad absorption bands in a lower-energy region, which can be ascribed to the ICT transitions from the donor to the acceptor units. The absorption edges for these transitions are located at 434 nm for **TB-BP-DMAC** and 389 nm for **TB-DMAC**. Considering that they have the same donor units, the significant difference in the absorption edges between these compounds should originate from their obviously different acceptor strengths. **TB-BP-DMAC** and **TB-DMAC** exhibit bright green and deep-blue photoluminescence (PL) in toluene with broad and structureless spectra peaking at 511 and 454 nm, and corresponding Commission Internationale de L’Eclairage (CIE) color coordinates of (0.28, 0.50) and (0.16, 0.16), respectively. The broad and structureless PL spectra confirm the ICT characteristics of the emissive states. The significant blue shift observed in the PL spectrum of **TB-DMAC**, when compared to that of **TB-BP-DMAC**, is likely attributed to the significantly weaker acceptor strength of the former.

To be consistent with the situation in the optimized OLEDs, the photophysical properties of these two emitters were further investigated in 20 wt%-doped 2,8-bis(diphenylphosphinyl)dibenzo[*b*,*d*]furan (PPF) films, in which PPF served as a host. Figure S10 demonstrates that the PL characteristics of these emitters in doped films are similar to those observed in toluene solutions. The steady-state PL spectra show a minor redshift with peak wavelengths at 517 and 467 nm and corresponding CIE color coordinates of (0.30, 0.49) and (0.18, 0.21) for **TB-BP-DMAC** and **TB-DMAC**, respectively. To gain a deeper understanding of the emissive states, we investigated the transient photoluminescence (PL) decay behavior of these emitters across various temperatures (Figure S13). Figure 2b illustrates that at room temperature, each emitter exhibits a transient PL curve comprising two distinct components: a prompt component and a delayed component. The prompt (τ_PF_) and delayed (τ_DF_) component lifetimes were fitted to be 12.1 ns and 2.55 μs for **TB-BP-DMAC** and 11.6 ns and 2.28 μs for **TB-DMAC**, respectively (Figures S11 and S12). The proportion of the DF component increases progressively as the temperature is elevated from 77 K to 300 K, indicating the typical TADF properties of both emitters (Figure S13). Therefore, it can be concluded that the delayed components observed in these emitters originate from TADF involving T_1_ → S_1_ → S_0_ transitions. To experimentally determine the S_1_ and T_1_ energy levels, as well as the energy gap (ΔE_ST_) between them, the time-resolved PL spectra of the doped film samples were acquired at 77 K (Figure 2c,d). The S_1_ energy, T_1_ energy, and ΔE_ST_ were calculated based on the onset wavelengths of the time-resolved PL spectra, yielding values of 2.81, 2.68, and 0.128 eV for **TB-BP-DMAC** and 3.12, 2.96, and 0.158 eV for **TB-DMAC**, respectively. The deep-blue emitter **TB-DMAC** demonstrates a relatively smaller ΔE_ST_ compared to the majority of previously reported deep-blue TADF emitters, while the green emitter **TB-BP-DMAC** exhibits a relatively larger ΔE_ST_ among green TADF emitters (Table S5). The small ΔE_ST_ values of these emitters allow an efficient up-conversion of excitons from the T_1_ state to the S_1_ state through RISC. Apparently, both the S_1_ and T_1_ states were effectively tuned by the substitution strategy. Directly linking the carbanyl groups with the nitrogen atoms on the Tröger’s base unit resulted in the formation of an amide acceptor, which exhibited a significantly weaker electron-deficient strength than that of the benzophenone acceptor and was found to be responsible for the observed changes in PL behaviors. The photoluminescence quantum yields (Φ_PL_) of **TB-BP-DMAC** and **TB-DMAC** were measured to be 31.5% and 50.4%, respectively. From Φ_PL_ and the respective proportions of the prompt and delayed fluorescence components in the transient PL decay curves, the quantum yields of prompt fluorescence (Φ_PF_) and delayed fluorescence (Φ_DF_) in doped films were calculated to be 21.2% and 10.3% for **TB-BP-DMAC** and 36.1% and 14.3% for **TB-DMAC**, respectively (Table 1). The rate constants of the key photophysical processes were calculated based on the efficiency and lifetime data by using a reported method (see Supporting Information for details). For **TB-BP-DMAC**, the rate constants follow the order of the non-radiative rate constant ($k_{nr}^{s}$) > intersystem-crossing (ISC) rate constants (k_ISC_) > the radiation rate constant ($k_{r}^{s}$). In contrast, for **TB-DMAC**, the order is $k_{r}^{s}$ > $k_{nr}^{s}$ > k_ISC_ (Table 1). The RISC rate constants (k_RISC_) of **TB-BP-DMAC** and **TB-DMAC** were calculated to be 5.83 × 10^5^ and 6.13 × 10^5^ s^−1^, respectively. These values suggest fast RISC processes in these molecules, which may be attributed to the small ΔE_ST_ values and the significant SOC interactions between the excited states involved in the RISC processes.

### 2.5. Electroluminescence Performance

To explore the electroluminescence (EL) properties of these TADF emitters, we fabricated multilayer doped OLEDs with a device configuration of ITO/HATCN (5 nm)/TAPC (30 nm)/TCTA (5 nm)/mCP (5 nm)/PPF: Emitter (30 nm)/PPF (10 nm)/Bphen (30 nm)/Liq (1 nm)/Al (100 nm), in which HATCN, TAPC, TCTA, mCP, PPF, Bphen, and Liq serve as the hole-injection layer, hole-transporting layer, electron-blocking layer, host, hole-blocking layer, electron-transporting layer, and electron-injecting layer, respectively. PPF was selected as a host material due to its high triplet energy (T_1_ = 3.1 eV), which is conducive to confine triplet excitons of the investigated emitters (T_1_ = 2.68–2.96 eV). The chemical structures of the functional organic materials used in the devices are shown in Figure S15.

**Figure 3:**
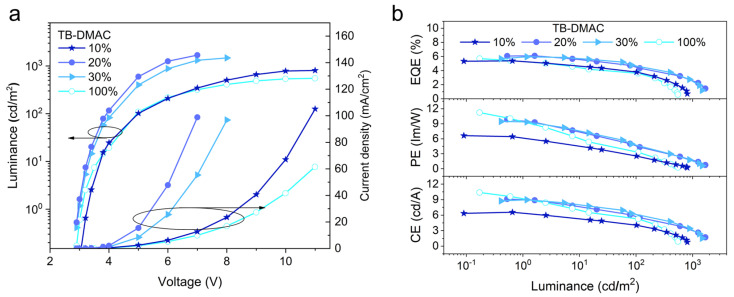
OLEDs based on **TB-DMAC** with various doping concentrations (in weight): (**a**) current density/voltage/luminance characteristics; (**b**) external quantum efficiency (EQE), power efficiency (PE), and current efficiency (CE) versus luminance characteristics.

At first, we investigated the dependence of EL performance on the doping concentration for **TB-DMAC**, using emitter doping concentrations of 10 wt%, 20 wt%, 30 wt%, and 100% (non-doped). The results are summarized in Figure 3 and Table 2. A slight spectral redshift in EL was observed as the doping concentration increased, with emission peaks ranging from 447 to 453 nm for **TB-DMAC** based devices when the doping concentration increased from 10% to 100% (Table 2). The **TB-DMAC** based device exhibited the highest EQE_max_ of 6.1% at a doping concentration of 20 wt%. Notably, while the doping concentration continued to increase to 30 wt% and 100% (non-doped), the EQEs did not decrease significantly. The non-doped device still maintained an EQE_max_ of 5.7%.

**Figure 4:**
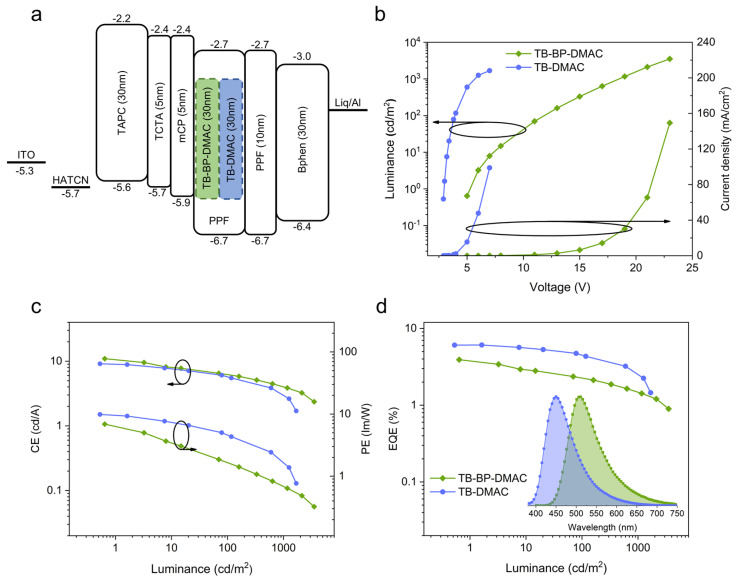
(**a**) Energy level diagram of the OLEDs; (**b**) current density/voltage/luminance characteristics; (**c**) current efficiency (CE)/luminance-power efficiency (PE) curves; (**d**) EL spectra (inset) of the devices and external quantum efficiency (EQE) vs. luminance relationships of devices.

The 20 wt% doped OLEDs employing **TB-BP-DMAC** as an emitter were also fabricated and characterized for comparison (Figure 4 and Table 2). The 20 wt% doped OLEDs based on **TB-BP-DMAC** and **TB-DAMC** turned on at 5.3 and 2.9 V and displayed green and deep-blue emissions with spectral peaks at 508 nm and 449 nm and CIE coordinates of (0.28, 0.51) and (0.17, 0.15), respectively. The **TB-DMAC**-based device exhibited a higher EQE_max_ of 6.1%, while the **TB-BP-DMAC**-based device achieved an EQE_max_ of only 3.9%, probably due to the lower PLQY.

## 3. Experimental Section

### 3.1. General Procedures

All air- and moisture-sensitive reactions were carried out under an argon atmosphere. All reagents and solvents were obtained commercially and used without further purification except as noted. Dry 1,4-dioxane, dry trifluoroacetic acid (TFA), dry tetrahydrofuran (THF), dry toluene, and dry dichloromethane (DCM) were purchased from Adamas Reagent, Ltd. (Shanghai, China). The three intermediates **TB-Br**, **TB-B**, and **TB** were prepared by procedures from the literature [43,44]. ^1^H NMR (500 MHz) and ^13^C NMR (126 MHz) spectra were recorded on a Bruker Avance III NMR spectrometer, in deuterated chloroform (CDCl_3_).

### 3.2. Synthesis of Materials

#### 3.2.1. Synthesis of ((4,10-Dimethyl-6*H*,12*H*-5,11-methanodibenzo[*b*,*f*][1,5]diazocine-2,8-diyl)bis(4,1-phenylene))bis((4-bromophenyl)methanone) (**TB-BP-Br**)

A mixture of **TB-B** (2.2 g, 4.5 mmol), bis(4-bromophenyl)methanone (4.59 g, 13.5 mmol), Pd(PPh_3_)_4_ (0.52 g, 0.45 mmol), THF (112.5 mL), and aqueous K_2_CO_3_ (1 M, 45 mL) was placed under vacuum and then backfilled with nitrogen three times before being heated in an oil bath at 60 °C for 16 h. After cooling to room temperature, the layers were separated. The aqueous phase was extracted with DCM (3 × 50 mL), the combined organic phases were dried over sodium sulfate and filtered, and then the solvent was removed. The residue was purified by column chromatography over silica using ethyl acetate/petroleum ether as eluent to afford **TB-BP-Br** as a white solid (1.15 g, yield: 33%). ^1^H NMR was as follows: (500 MHz, CDCl_3_) δ 7.81 (d, *J* = 8.3 Hz, 4H), 7.68–7.54 (m, 12H), 7.38 (s, 2H), 7.10 (s, 2H), 4.74 (d, *J* = 13.8 Hz, 2H), 4.45 (s, 2H), 4.16 (d, *J* = 16.8 Hz, 2H), and 2.53 (s, 6H).

#### 3.2.2. Synthesis of (4,10-Dimethyldibenzo[*b*,*f*][1,5]diazocine-5,11(6*H*,12*H*)-diyl)bis((4-bromophenyl)methanone) (**TB-PhBr**)

Aluminum trichloride (1.60 g, 12 mmol), **TB** (1.25 g, 5 mmol), and DCM (50 mL) were added to the flask under vacuum and then backfilled with nitrogen three times at 0 °C. After stirring for 15 min, a solution of 4-bromobenzoyl chloride (2.42 g, 11 mmol) was added dropwise under stirring. The reaction was performed for three hours at 40 °C. Dilute hydrochloric acid was slowly added dropwise to the flask to terminate the reaction. The reaction was extracted with dichloromethane (3 × 40 mL). After collecting organic-phase, it was dried with anhydrous Na_2_SO_4_, the solvent was removed to give the crude product. Using petroleum ether and dichloromethane-mixed solvent as eluent, silica gel column separation and purification were performed, followed by vacuum-drying to obtain a white solid (1.51 g, yield: 50%). ^1^H NMR was as follows: (500 MHz, CDCl_3_) δ 7.29 (ddd, *J* = 10.7, 5.3, 2.1 Hz, 7H), 7.26 (d, *J* = 2.0 Hz, 1H), 6.96–6.90 (m, 4H), 6.83 (dd, *J* = 6.7, 2.2 Hz, 2H), 5.70 (d, *J* = 14.9 Hz, 2H), 4.39 (d, *J* = 15.0 Hz, 2H), and 2.13 (s, 6H).

#### 3.2.3. Synthesis of ((4,10-dimethyl-6H,12H-5,11-methanodibenzo[*b,f*][1,5]diazocine-2,8-diyl)bis(4,1-phenylene))bis((4-(9,9-dimethylacridin-10(9H)-yl)phenyl)methanone) (**TB-BP-DMAC**)

A mixture of **TB-BP-Br** (1.15 g, 1.5 mmol), 9,9-dimethyl-9,10-dihydroacridine (0.94 g, 4.5 mmol), tri-tert-butylphosphine tetrafluoroborate (0.087 g, 0.3 mmol), sodium tert-butoxide (0.43 g, 4.5 mmol), palladium acetate (0.035 g, 0.15 mmol), and dry toluene was placed under vacuum and then backfilled with nitrogen three times before being heated in an oil bath at 110 °C for 18 h. After cooling to room temperature, the solvent was removed by vacuum rotary evaporation. The mixture was extracted with dichloromethane (3 × 40 mL). After collecting organic-phase, it was dried with anhydrous Na_2_SO_4_, the solvent was removed to give the crude product. Using petroleum ether and dichloromethane-mixed solvent as eluent, silica gel column separation and purification were performed, followed by vacuum drying to obtain a yellow-green solid (1.38 g, yield 90%). _1_H NMR was as follows: (500 MHz, CDCl_3_) δ 8.11–8.04 (m, 4H), 7.95 (d, *J* = 8.4 Hz, 4H), 7.67 (d, *J* = 8.4 Hz, 4H), 7.54–7.45 (m, 8H), 7.41 (s, 2H), 7.14 (s, 2H), 6.99 (dtd, *J* = 21.2, 7.3, 1.4 Hz, 8H), 6.34 (dd, *J* = 8.0, 1.3 Hz, 4H), 4.75 (s, 2H), 4.46 (s, 2H), 4.19 (d, *J* = 16.6 Hz, 2H), 2.55 (s, 6H), and 1.70 (s, 12H). ^13^C NMR was as follows: (126 MHz, CDCl_3_) 195.42, 145.30, 140.49, 137.23, 135.64, 133.75, 132.60, 130.95, 130.74, 130.60, 129.98, 128.30, 126.77, 126.43, 125.37, 123.43, 121.09, 114.33, 55.20, 36.09, 31.13, and 17.45.

#### 3.2.4. Synthesis of (4,10-dimethyldibenzo[*b,f*][1,5]diazocine-5,11(6H,12H)-diyl)bis((4-(9,9-dimethylacridin-10(9H)-yl)phenyl)methanone) (**TB-DMAC**)

It was prepared by the same procedure as that of **TB-BP-DMAC**, except **TB-PhBr** was used instead of **TB-BP-Br**. Vacuum drying was performed to obtain a white solid (yield: 85%). ^1^H NMR was as follows: (500 MHz, CDCl_3_) δ 7.83 (d, *J* = 7.3 Hz, 2H), 7.47 (d, *J* = 8.4 Hz, 4H), 7.41 (dd, *J* = 5.9, 3.3 Hz, 4H), 7.29 (t, *J* = 7.6 Hz, 2H), 7.11 (t, *J* = 7.9 Hz, 6H), 6.95–6.82 (m, 8H), 6.01 (dd, *J* = 6.0, 3.4 Hz, 4H), 5.74 (d, *J* = 13.3 Hz, 2H), 4.44 (d, *J* = 13.3 Hz, 2H), 2.16 (s, 6H), and 1.61 (d, *J* = 9.2 Hz, 12H). ^13^C NMR was as follows: (126 MHz, CDCl_3_) 168.84, 144.27, 143.08, 140.40, 135.02, 134.89, 134.80, 131.74, 131.08, 130.74, 130.44, 130.27, 128.37, 126.27, 125.18, 120.85, 113.86, 54.77, 35.97, 30.94, and 18.36.

### 3.3. Thermogravimetric Analyses

Thermogravimetric analyses were performed on a METTLER TOLEDO system (METTLER-TOLEDO International Inc., Columbus, Switzerland) with a heating rate of 10 °C/min under nitrogen.

### 3.4. Electrochemical Measurement

Cyclic voltammetry was performed at room temperature in anhydrous and argon-saturated dichloromethane solutions of 0.1 M tetrabutylammonium hexafluorophosphate and 1.0 mM investigated compounds with a CHI840D electrochemical analyzer (CH Instruments, Inc., Austin, TX, USA). Glassy carbon, platinum wire, and Ag/Ag^+^ (0.01 M of AgNO_3_ in acetonitrile) were selected as the working electrode, auxiliary electrode, and reference electrode, respectively. The ferrocenium/ferrocene couple was used as an internal standard.

### 3.5. Photophysical Measurements

UV–Vis absorption spectra were recorded with an Agilent Cary 5000 (Agilent Technologies, Santa Clara, CA, USA) UV–Vis spectrophotometer under ambient conditions. The absolute PLQYs were measured on an Edinburgh F900 spectrophotometer (Edinburgh Instruments Limited, Edinburgh, United Kingdom) equipped with an integrating sphere with the followsing properties: reference sample: BaSO_4_; film samples: 20 wt% of **TB-BP-DMAC** or **TB-DMAC** doped into PPF host, prepared by vacuum deposition; and excitation wavelength: 300 nm. The excitation spectra and emission spectra were collected in the wavelength ranges of 285–315 nm and 360–750 nm, respectively. Steady-state PL spectra were measured on an Edinburgh FLS980 (Edinburgh Instruments Limited, Edinburgh, UK) using a Xenon lamp as an excitation light source. The transient PL decay curves were measured on the same spectrophotometer (FLS980) in time-correlated single-photon counting (TCSPC) mode with an NT242-1K OPO laser (Ekspla, Vilnius, Lithuania) as an excitation light source. The time-resolved PL spectra were recorded on an Edinburgh LP980 spectrophotometer with an NT242-1K OPO laser excitation source.

### 3.6. Computational Methodology

All the calculations were carried out using the Gaussian 09 program package. The density functional theory (DFT) calculations at the BMK/6-31G** level were used to optimize the ground state geometries of the investigated compounds. Time-dependent density functional theory (TD-DFT) calculations were performed at the same level using the optimized ground state geometries. The spin–orbit couplings were calculated at B3LYP/6-31G** by ORCA. The electron density diagrams of molecular orbitals were generated using GaussView program. The partition orbital composition was analyzed by using the Multiwfn 2.4 program.

### 3.7. Device Fabrication and Characterization

Indium tin oxide (ITO) glass substrates were cleaned successively in an ultrasonic bath containing deionized water, acetone, and isopropanol. They were then blown dry with N_2_ and treated in UV/ozone ambient conditions for 15 min prior to film deposition. The organic materials were deposited onto the ITO-coated substrates at a rate of 1 Å s^−1^ under high vacuum (<1 × 10^−4^) by thermal evaporation in vacuum chamber. Then Liq and Al were successively deposited at a rate of 0.1 Å s^−1^ and 5 Å s^−1^, respectively. The electroluminescence (EL) spectra and Commission Internatinale de L’Eclairage coordinates (CIE) of the OLEDs were recorded by an integrated optoelectronic performance test system with a calibrated spectra radiometer (TOPCON SR-UL1R) (Topcon Corporation, Tokyo, Japan). The current efficiency and power efficiency were measured using Keithley 2400 source meter (Tektronix, Inc., Beaverton, OR, USA).

## 4. Conclusions

In summary, we have designed and synthesized two TADF emitters with D-A-D molecular configuration, named **TB-BP-DMAC** and **TB-DMAC**, by employing Tröger’s-base-fused benzophenone and amide acceptors as the electron acceptors, respectively. Our theoretical and experimental investigations revealed that the amide acceptor in **TB-DMAC** exhibits a much weaker strength compared to the benzophenone acceptor used in **TB-BP-DMAC**, which results in a significantly blue-shifted emission from green to deep blue, a higher emission efficiency, and a faster RISC process. **TB-BP-DMAC** and **TB-DMAC** exhibit green and deep-blue photoluminescence with PLQYs of 31.5% and 50.4% as well as decay fluorescence lifetimes of 2.55 and 2.28 μs in doped films, respectively. By using **TB-DMAC** as emitter, the doped and non-doped OLEDs have efficient deep-blue electroluminescence peaks at 449 and 453 nm with maximum EQEs of 6.1% and 5.7%, respectively, which far exceed the device performances of **TB-BP-DMAC**-based green-emitting devices. This indicates that substituted amide acceptors are a viable option for the design of high-performance deep-blue TADF materials. These results demonstrate the high potential of substituted amide acceptors in the construction of high-performance deep-blue TADF materials.

## Figures and Tables

**Table 1:** Photophysical data of the investigated compounds in 20 wt%-doped PPF film.

Compound	λ_PL_^a^ [nm]	Φ_PL_^b^ [%]	Φ_PF_/Φ_DF_^c^ [%]	τ_PF_/τ_DF_^d^ [ns/μs]	E_S1_/E_T1_/ΔE_ST_^e^ [eV]	$k_{r}^{s}$$/k_{nr}^{s}$/k_ISC_^f^ [10^7^s^−1^]	k_RISC_^g^ [10^5^s^−1^]	CIE_1931_ [x,y]
**TB-BP-DMAC**	517	31.5	21.2/10.3	12.1/2.55	2.81/2.68/0.128	1.75/3.81/2.69	5.83	(0.30, 0.49)
**TB-DMAC**	467	50.4	36.1/14.3	11.6/2.28	3.12/2.96/0.158	3.11/3.06/2.43	6.13	(0.18, 0.21)

^a^ the wavelength at photoluminescence maximum; ^b^ overall PLQY; ^c^ Φ_PF_ and Φ_DF_ are the quantum yields of prompt fluorescence and delayed fluorescence, respectively; ^d^ τ_PF_ and τ_DF_ are the lifetimes of prompt fluorescence and delayed fluorescence, respectively; ^e^ energy levels of S_1_ and T_1_ state were estimated from the onsets of time-resolved PL spectra at 77 K (Figure 2c,d); ^f^ $k_{r}^{s}$, $k_{nr}^{s}$, and k_ISC_ represent the radiative, nonradiative rate constant, and intersystem-crossing (ISC) rate constant of the S_1_ states, respectively; ^g^ k_RISC_ refers to the rate constants of reverse ISC.

**Table 2:** Summary of device performances.

Device	Doping Concentration	λ_EL_^a^ [nm]	V_on_^b^ [V]	L_max_^c^ [cd/m^2^]	EQE_max_^d^ [%]	CE_max_^e^ [cd/A]	PE_max_^f^ [lm/W]	CIE_1931_^g^ [x,y]
**TB-DMAC**	10%	447	3.2	806	5.4	6.6	6.6	(0.17, 0.13)
	20%	449	2.9	1684	6.1	9.1	9.9	(0.17, 0.15)
	30%	452	3.0	1474	6.0	9.0	9.5	(0.17, 0.16)
	100% (non-doped)	453	3.1	552	5.7	10.4	11.2	(0.18, 0.19)
**TB-BP-DMAC**	20%	508	5.3	3524	3.9	11.0	6.9	(0.28, 0.51)

^a^ the wavelength at electroluminescence maximum (recorded at 8 V); ^b^ turn-on voltage at 1 cd/m^2^; ^c^ maximum luminance; ^d^ EQE maximum value; ^e^ current efficiency maximum value; ^f^ power efficiency maximum value; ^g^ Commission Internatinale de L’Eclairage coordinates measured at 8 V.

## Data Availability

All the data generated by this research are included in the article.

## Supplementary Materials

The following supporting information can be downloaded at: https://www.mdpi.com/article/10.3390/molecules28124832/s1, Figure S1: ^1^H-NMR spectrum of **TB-BP-Br** (500 MHz, CDCl_3_); Figure S2. ^1^H-NMR spectrum of **TB-PhBr** (500 MHz, CDCl_3_); Figure S3. ^1^H-NMR spectrum of **TB-BP-DMAC** (500 MHz, CDCl_3_); Figure S4. ^13^C-NMR spectrum of **TB-BP-DMAC** (126 MHz, CDCl_3_); Figure S5. ^1^H-NMR spectrum of **TB-DMAC** (500 MHz, CDCl_3_); Figure S6. ^13^C-NMR spectrum of **TB-DMAC** (126 MHz, CDCl_3_); Figure S7. TGA curves of **TB-BP-DMAC** and **TB-DMAC** (The red dashed line marks 95% of the original sample weight); Figure S8. Cyclic Voltammograms of **TB-BP-DMAC** and **TB-DMAC**. Dashed vertical lines and intersections of tangent lines indicate the E_ox_ values; Figure S9. Electron-density distribution of the excited states (the purple area denotes an increase in charge density, while the blue area denotes a decrease in charge density) and character; Figure S10. PL spectra (20 wt%-doped PPF films) measured at room temperature; Figure S11. Transient PL decay curves (0–125 ns time-range, prompt decay) of (a) **TB-BP-DMAC** and (b) **TB-DMAC** in 20 wt%-doped PPF film at room temperature. Inset shows the fitting function along with the fitting results for the lifetimes of the prompt component, as well as the instrumental response function (IRF); Figure S12. Transient PL decay curves of (a) **TB-BP-DMAC** and (b) **TB-DMAC** in 20 wt%-doped PPF film at room temperature. Inset shows the fitting function and fitting results of delayed component lifetimes; Figure S13. Transient PL decay curves of (a) **TB-BP-DMAC** and (b) **TB-DMAC** in 20 wt%-doped PPF film at different temperatures; Figure S14. The comparison of EL (20 wt%-doped OLEDs) and PL spectra (20 wt%-doped films) of **TB-BP-DMAC** and **TB-DAMC**. The EL spectra were recorded at 8 V. The PL spectra were obtained using 300 nm excitation. All spectra were obtained at room temperature; Figure S15. Molecular structures of the functional materials used in the OLEDs; Table S1: Summary of CV data and energy levels; Table S2. Calculated spin-orbit coupling (SOC) constants; Table S3. Fitting results of the transient PL decay curves of prompt fluorescence; Table S4. Fitting results of the transient PL decay curves of delayed fluorescence; Table S5. Summary of ΔE_ST_ values for some deep blue and green emitters. References [38,45,46,47,48,49,50,51] are cited in the supplementary materials.

## Sample Availability

Samples of the compounds are not available from the authors.

## References

[1] Tang C.W., Vanslyke S.A. (1987). Organic electroluminescent diodes. Appl. Phys. Lett..

[2] Baldo M.A., O’Brien D.F., You Y., Shoustikov A., Sibley S., Thompson M.E., Forrest S.R. (1998). Highly efficient phosphorescent emission from organic electroluminescent devices. Nature.

[3] Uoyama H., Goushi K., Shizu K., Nomura H., Adachi C. (2012). Highly efficient organic light-emitting diodes from delayed fluorescence. Nature.

[4] Song W., Lee J.Y. (2017). Degradation Mechanism and Lifetime Improvement Strategy for Blue Phosphorescent Organic Light-Emitting Diodes. Adv. Opt. Mater..

[5] Sivasubramaniam V., Brodkorb F., Hanning S., Loebl H.P., van Elsbergen V., Boerner H., Scherf U., Kreyenschmidt M. (2009). Fluorine cleavage of the light blue heteroleptic triplet emitter FIrpic. J. Fluor. Chem..

[6] Udagawa K., Sasabe H., Cai C., Kido J. (2014). Low-driving-voltage blue phosphorescent organic light-emitting devices with external quantum efficiency of 30%. Adv. Mater..

[7] Chen X.K., Kim D., Bredas J.L. (2018). Thermally Activated Delayed Fluorescence (TADF) Path toward Efficient Electroluminescence in Purely Organic Materials: Molecular Level Insight. Acc. Chem. Res..

[8] Adachi C. (2014). Third-generation organic electroluminescence materials. Jpn. J. Appl. Phys..

[9] Wong M.Y., Zysman-Colman E. (2017). Purely Organic Thermally Activated Delayed Fluorescence Materials for Organic Light-Emitting Diodes. Adv. Mater..

[10] Dias F.B., Penfold T.J., Monkman A.P. (2017). Photophysics of thermally activated delayed fluorescence molecules. Methods Appl. Fluoresc..

[11] Cui L.-S., Gillett A.J., Zhang S.-F., Ye H., Liu Y., Chen X.-K., Lin Z.-S., Evans E.W., Myers W.K., Ronson T.K. (2020). Fast spin-flip enables efficient and stable organic electroluminescence from charge-transfer states. Nat. Photonics.

[12] Ji S.-C., Zhao T., Wei Z., Meng L., Tao X.-D., Yang M., Chen X.-L., Lu C.-Z. (2022). Manipulating excited states via Lock/Unlock strategy for realizing efficient thermally activated delayed fluorescence emitters. Chem. Eng. J..

[13] He J.-L., Kong F.-C., Sun B., Wang X.-J., Tian Q.-S., Fan J., Liao L.-S. (2021). Highly efficient deep-red TADF organic light-emitting diodes via increasing the acceptor strength of fused polycyclic aromatics. Chem. Eng. J..

[14] Godumala M., Choi S., Cho M.J., Choi D.H. (2016). Thermally activated delayed fluorescence blue dopants and hosts: From the design strategy to organic light-emitting diode applications. J. Mater. Chem. C.

[15] Cai X., Su S.-J. (2018). Marching Toward Highly Efficient, Pure-Blue, and Stable Thermally Activated Delayed Fluorescent Organic Light-Emitting Diodes. Adv. Funct. Mater..

[16] Im Y., Byun S.Y., Kim J.H., Lee D.R., Oh C.S., Yook K.S., Lee J.Y. (2017). Recent Progress in High-Efficiency Blue-Light-Emitting Materials for Organic Light-Emitting Diodes. Adv. Funct. Mater..

[17] Bui T.T., Goubard F., Ibrahim-Ouali M., Gigmes D., Dumur F. (2018). Recent advances on organic blue thermally activated delayed fluorescence (TADF) emitters for organic light-emitting diodes (OLEDs). Beilstein J. Org. Chem..

[18] Bui T.-T., Goubard F., Ibrahim-Ouali M., Gigmes D., Dumur F. (2018). Thermally Activated Delayed Fluorescence Emitters for Deep Blue Organic Light Emitting Diodes: A Review of Recent Advances. Appl. Sci..

[19] Lee J.-H., Chen C.-H., Lee P.-H., Lin H.-Y., Leung M.-k., Chiu T.-L., Lin C.-F. (2019). Blue organic light-emitting diodes: Current status, challenges, and future outlook. J. Mater. Chem. C.

[20] Liu Y., Li C., Ren Z., Yan S., Bryce M.R. (2018). All-organic thermally activated delayed fluorescence materials for organic light-emitting diodes. Nat. Rev. Mater..

[21] Jeon S.K., Lee H.L., Yook K.S., Lee J.Y. (2019). Recent Progress of the Lifetime of Organic Light-Emitting Diodes Based on Thermally Activated Delayed Fluorescent Material. Adv. Mater..

[22] Jang H.J., Lee J.Y., Kim J., Kwak J., Park J.-H. (2020). Progress of display performances: AR, VR, QLED, and OLED. J. Inf. Disp..

[23] Gao Y., Wu S., Shan G., Cheng G. (2022). Recent Progress in Blue Thermally Activated Delayed Fluorescence Emitters and Their Applications in OLEDs: Beyond Pure Organic Molecules with Twist D-π-A Structures. Micromachines.

[24] Shi C., Liu D., Li J., He Z., Song K., Liu B., Wu Q., Xu M. (2022). tert-Butyltriazine-Diphenylaminocarbazole based TADF materials: π-Bridge modification for enhanced kRISC and efficiency stability. Dyes Pigm..

[25] Konidena R.K., Lee J.Y. (2019). Molecular Design Tactics for Highly Efficient Thermally Activated Delayed Fluorescence Emitters for Organic Light Emitting Diodes. Chem. Rec..

[26] Che W., Xie Y., Li Z. (2020). Structural Design of Blue-to-Red Thermally-Activated Delayed Fluorescence Molecules by Adjusting the Strength between Donor and Acceptor. Asian J. Org. Chem..

[27] Huang W., Xie L., Feng Q., Wang H., Qian Y., Zhou T. (2021). Recent Advances in Substituent Effects of Blue Thermally Activated Delayed Fluorescence Small Molecules. Acta Chim. Sin..

[28] Zhao W., He Z., Lam J.W.Y., Peng Q., Ma H., Shuai Z., Bai G., Hao J., Tang B.Z. (2016). Rational Molecular Design for Achieving Persistent and Efficient Pure Organic Room-Temperature Phosphorescence. Chem.

[29] Yun J.H., Lee K.H., Lee J.Y. (2021). Benzoylphenyltriazine as a new acceptor of donor–acceptor type thermally-activated delayed-fluorescent emitters. J. Ind. Eng. Chem..

[30] Hu J., Zhang X., Zhang D., Cao X., Jiang T., Zhang X., Tao Y. (2017). Linkage modes on phthaloyl/triphenylamine hybrid compounds: Multi-functional AIE luminogens, non-doped emitters and organic hosts for highly efficient solution-processed delayed fluorescence OLEDs. Dyes Pigm..

[31] Matsuoka K., Albrecht K., Nakayama A., Yamamoto K., Fujita K. (2018). Highly Efficient Thermally Activated Delayed Fluorescence Organic Light-Emitting Diodes with Fully Solution-Processed Organic Multilayered Architecture: Impact of Terminal Substitution on Carbazole-Benzophenone Dendrimer and Interfacial Engineering. ACS Appl. Mater. Interfaces.

[32] Yang Y., Wang S., Zhu Y., Wang Y., Zhan H., Cheng Y. (2018). Thermally Activated Delayed Fluorescence Conjugated Polymers with Backbone-Donor/Pendant-Acceptor Architecture for Nondoped OLEDs with High External Quantum Efficiency and Low Roll-Off. Adv. Funct. Mater..

[33] Wu L., Wang K., Wang C., Fan X.C., Shi Y.Z., Zhang X., Zhang S.L., Ye J., Zheng C.J., Li Y.Q. (2020). Using fluorene to lock electronically active moieties in thermally activated delayed fluorescence emitters for high-performance non-doped organic light-emitting diodes with suppressed roll-off. Chem. Sci..

[34] Zou S.N., Peng C.C., Yang S.Y., Qu Y.K., Yu Y.J., Chen X., Jiang Z.Q., Liao L.S. (2021). Fully Bridged Triphenylamine Derivatives as Color-Tunable Thermally Activated Delayed Fluorescence Emitters. Org. Lett..

[35] Huang F., Wang K., Shi Y.Z., Fan X.C., Zhang X., Yu J., Lee C.S., Zhang X.H. (2021). Approaching Efficient and Narrow RGB Electroluminescence from D-A-Type TADF Emitters Containing an Identical Multiple Resonance Backbone as the Acceptor. ACS Appl. Mater. Interfaces.

[36] Fan X.C., Wang K., Shi Y.Z., Chen J.X., Huang F., Wang H., Hu Y.N., Tsuchiya Y., Ou X.M., Yu J. (2021). Managing Intersegmental Charge-Transfer and Multiple Resonance Alignments of D-A Typed TADF Emitters for Red OLEDs with Improved Efficiency and Color Purity. Adv. Opt. Mater..

[37] Huang J., Nie H., Zeng J., Zhuang Z., Gan S., Cai Y., Guo J., Su S.J., Zhao Z., Tang B.Z. (2017). Highly Efficient Nondoped OLEDs with Negligible Efficiency Roll-Off Fabricated from Aggregation-Induced Delayed Fluorescence Luminogens. Angew. Chem. Int. Ed..

[38] Wu X., Zeng J., Peng X., Liu H., Tang B.Z., Zhao Z. (2023). Robust sky-blue aggregation-induced delayed fluorescence materials for high-performance top-emitting OLEDs and single emissive layer white OLEDs. Chem. Eng. J..

[39] Liu H., Fu Y., Tang B.Z., Zhao Z. (2022). All-fluorescence white organic light-emitting diodes with record-beating power efficiencies over 130 lm W^−1^ and small roll-offs. Nat. Commun..

[40] Cai M., Auffray M., Zhang D., Zhang Y., Nagata R., Lin Z., Tang X., Chan C.-Y., Lee Y.-T., Huang T. (2021). Enhancing spin-orbital coupling in deep-blue/blue TADF emitters by minimizing the distance from the heteroatoms in donors to acceptors. Chem. Eng. J..

[41] Rúnarsson Ö.V., Artacho J., Wärnmark K. (2012). The 125th Anniversary of the Tröger’s Base Molecule: Synthesis and Applications of Tröger’s Base Analogues. Eur. J. Org. Chem..

[42] Neogi I., Jhulki S., Ghosh A., Chow T.J., Moorthy J.N. (2015). Amorphous host materials based on Troger’s base scaffold for application in phosphorescent organic light-emitting diodes. ACS Appl. Mater. Interfaces.

[43] Kiehne U., Bruhn T., Schnakenburg G., Frohlich R., Bringmann G., Lutzen A. (2008). Synthesis, resolution, and absolute configuration of difunctionalized Troger’s base derivatives. Chemistry.

[44] Didier D., Tylleman B., Lambert N., Vande Velde C.M.L., Blockhuys F., Collas A., Sergeyev S. (2008). Functionalized analogues of Tröger’s base: Scope and limitations of a general synthetic procedure and facile, predictable method for the separation of enantiomers. Tetrahedron.

[45] Wada Y., Nakagawa H., Matsumoto S., Wakisaka Y., Kaji H. (2020). Organic light emitters exhibiting very fast reverse intersystem crossing. Nat. Photonics.

[46] Chen X.-L., Tao X.-D., Wei Z., Meng L., Lin F.-L., Zhang D.-H., Jing Y.-Y., Lu C.-Z. (2021). Thermally Activated Delayed Fluorescence Amorphous Molecular Materials for High-Performance Organic Light-Emitting Diodes. ACS Appl. Mater. Interfaces.

[47] Gao H., Shen S., Qin Y., Liu G., Gao T., Dong X., Pang Z., Xie X., Wang P., Wang Y. (2022). Ultrapure Blue Thermally Activated Delayed Fluorescence (TADF) Emitters Based on Rigid Sulfur/Oxygen-Bridged Triarylboron Acceptor: MR TADF and D-A TADF. J. Phys. Chem. Lett..

[48] Liu B., Li J., Liu D., Mei Y., Lan Y., Song K., Li Y., Wang J. (2022). Electron-withdrawing bulky group substituted carbazoles for blue TADF emitters: Simultaneous improvement of blue color purity and RISC rate constants. Dyes Pigm..

[49] Lee Y.H., Park S., Oh J., Shin J.W., Jung J., Yoo S., Lee M.H. (2017). Rigidity-Induced Delayed Fluorescence by Ortho Donor-Appended Triarylboron Compounds: Record-High Efficiency in Pure Blue Fluorescent Organic Light-Emitting Diodes. ACS Appl. Mater. Interfaces.

[50] Xia Y., Li J., Chen X., Li A., Guo K., Chen F., Zhao B., Chen Z., Wang H. (2022). Molecular Engineering of Push-Pull Diphenylsulfone Derivatives towards Aggregation-Induced Narrowband Deep Blue Thermally Activated Delayed Fluorescence (TADF) Emitters. Chemistry.

[51] Yoo J.-Y., Choi Y.J., Kim K.W., Ha T.H., Lee C.W. (2023). Improved device efficiency and lifetime of green thermally activated delayed fluorescence materials with multiple donors and cyano substitution. Dyes Pigm..

