# Supplementary material for: Tröger’s Base-Derived Thermally Activated Delayed Fluorescence Dopant for Efficient Deep-Blue Organic Light-Emitting Diodes
Source: Molecules. 2023 Jun 17;28(12):4832. doi: 10.3390/molecules28124832 (PMC10304498; doi:10.3390/molecules28124832)
Supplement: Supplementary file 1 [file molecules-28-04832-s001.zip › molecules-2399745-supplementary.pdf]

Supporting Information

Tröger's Base-Derived Thermally Activated Delayed Fluorescence Dopant for Efficient Deep-Blue Organic Light-Emitting Diodes

Ze-Ling Wu [1,2,3,4], Xin Lv [1,2,3], Ling-Yi Meng [1,2,3], Xu-Lin Chen [1,2,3,*] and Can-Zhong Lu [1,2,3,4,*]

[1] State Key Laboratory of Structural Chemistry, Fujian Institute of Research on the Structure of Matter, Chinese Academy of Sciences, Fuzhou 350002, China; wuzeling20@mails.ucas.ac.cn (Z.-L.W.); xmlvxin@fjirsm.ac.cn (X.L.); lymeng@fjirsm.ac.cn (L.-Y.M.)
[2] Fujian Science & Technology Innovation Laboratory for Optoelectronic Information of China, Fuzhou 350108, China
[3] Xiamen Key Laboratory of Rare Earth Photoelectric Functional Materials, Xiamen Institute of Rare Earth Materials, Haixi Institutes, Chinese Academy of Sciences, Xiamen 361021, China
[4] University of Chinese Academy of Sciences, Beijing 100049, China
* Correspondence: xlchem@fjirsm.ac.cn (X.-L.C.); czlu@fjirsm.ac.cn (C.-Z.L.)

Table of Contents

1. NMR Spectra S2
2. Thermogravimetric Analysis (TGA)..S5
3. Electrochemical Properties S6
4. Theoretical Calculations S8
5. Photophysical Properties..S8
6. Device Fabrication and Characterization..S15
7. Reference S16

1. NMR Spectra

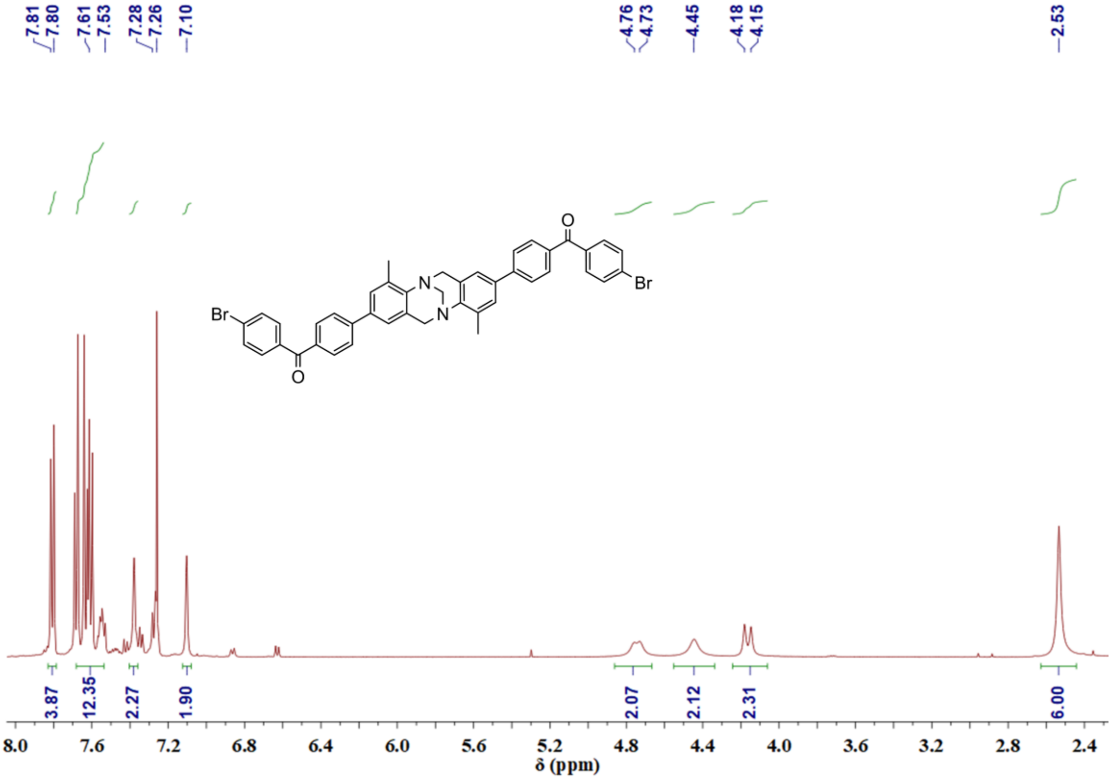

Figure S1. ^{1}H-NMR spectrum of **TB-BP-Br** (500 MHz, $CDCl_3$).

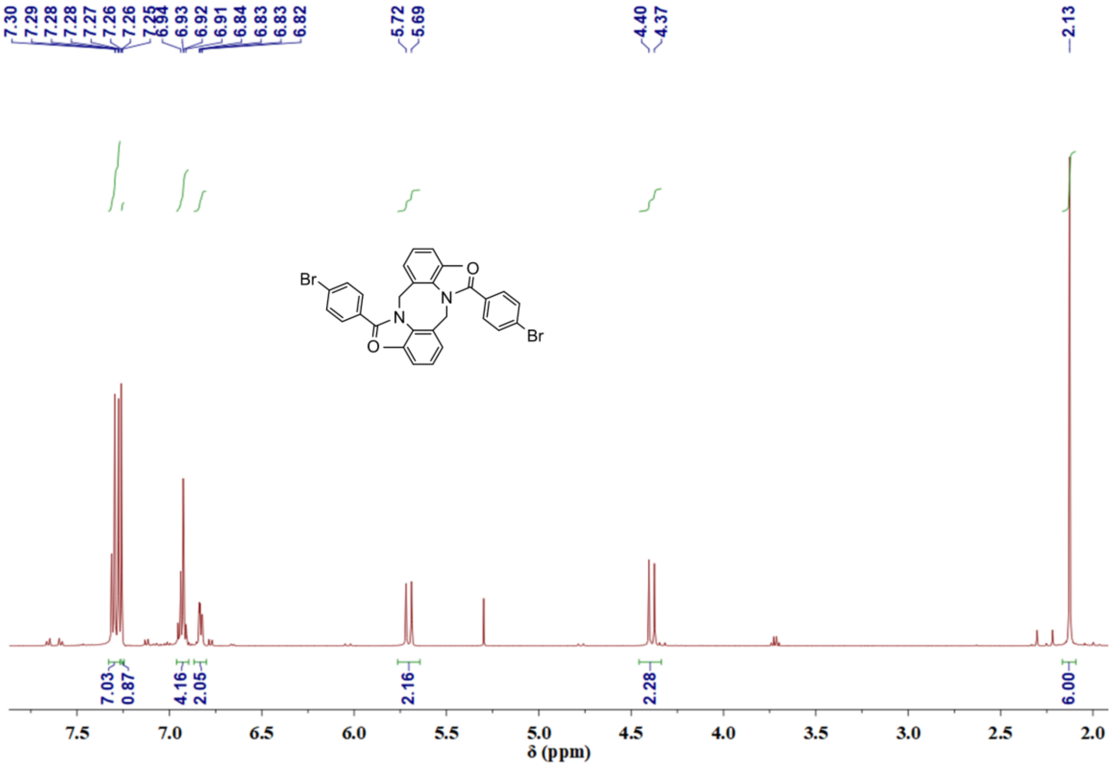

Figure S2. ^{1}H-NMR spectrum of **TB-PhBr** (500 MHz, $CDCl_3$).

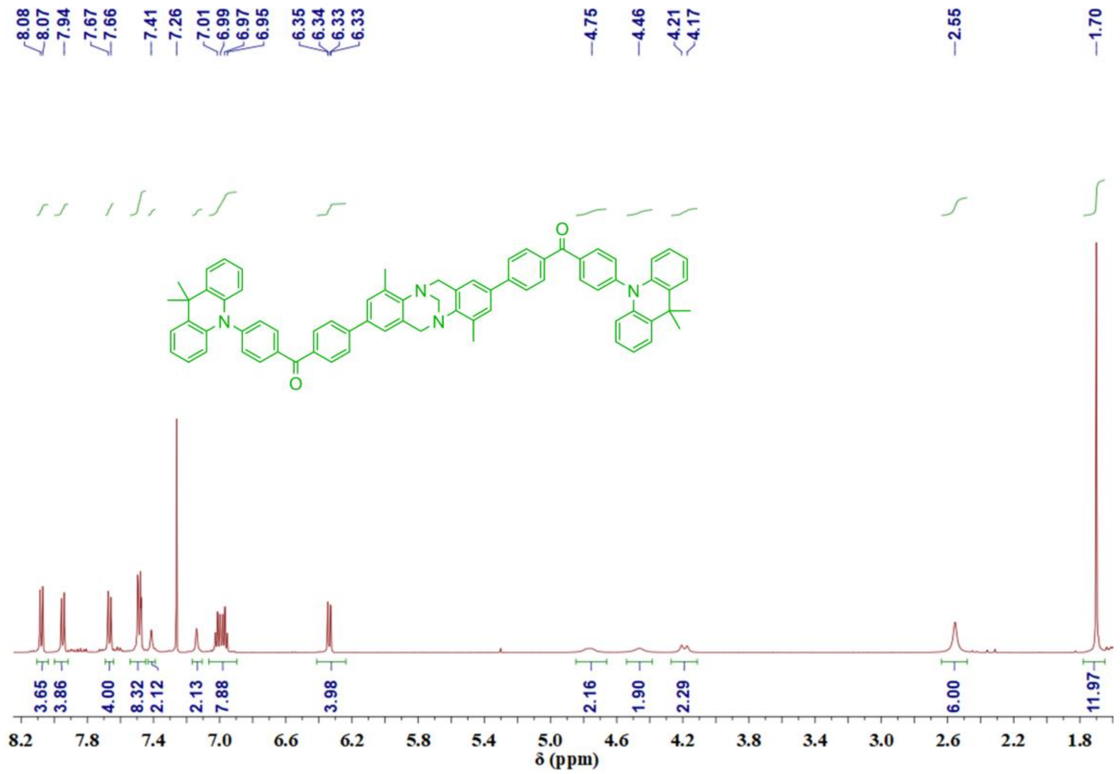

Figure S3. ^{1}H-NMR spectrum of **TB-BP-DMAC** (500 MHz, $CDCl_3$).

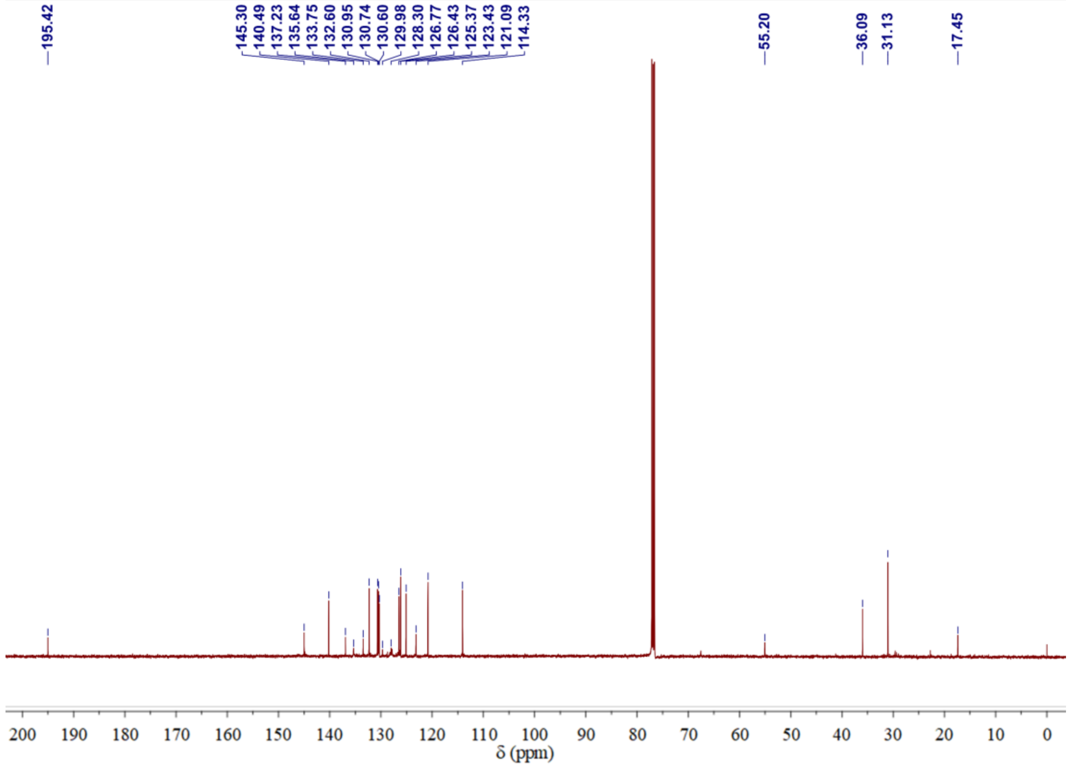

Figure S4. ^{13}C-NMR spectrum of **TB-BP-DMAC** (126 MHz, $CDCl_3$).

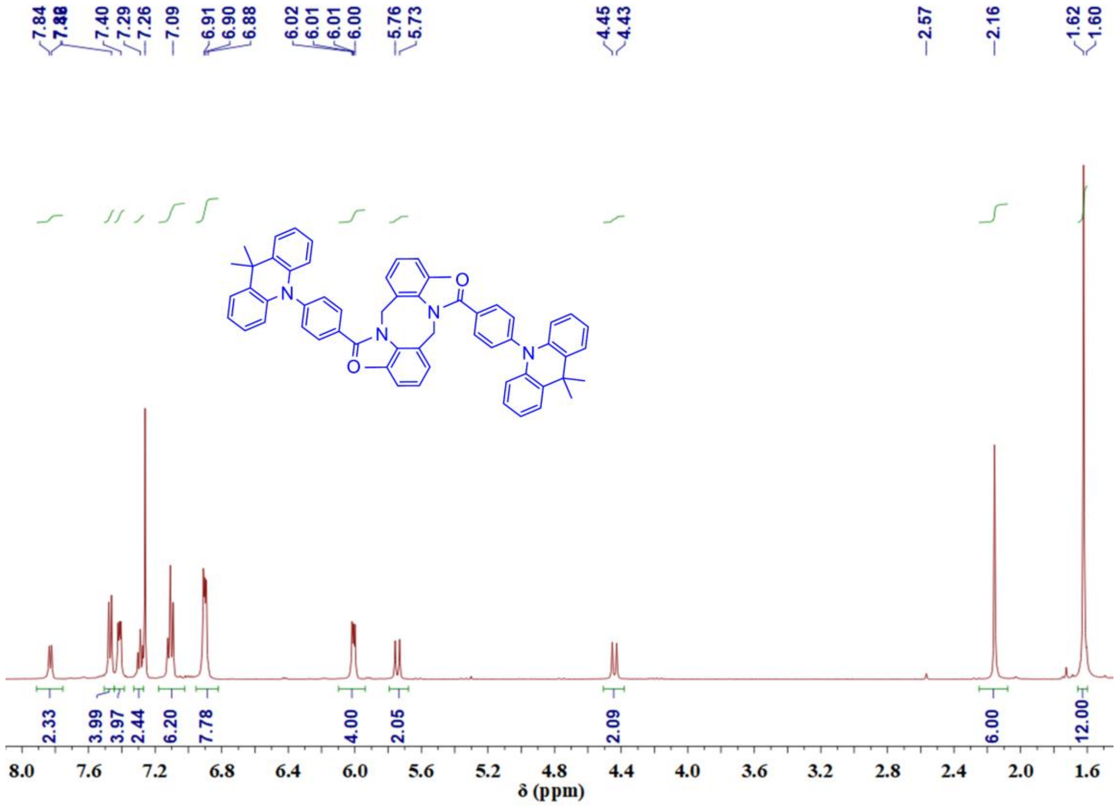

Figure S5. ^{1}H-NMR spectrum of **TB-DMAC** (500 MHz, $CDCl_3$)

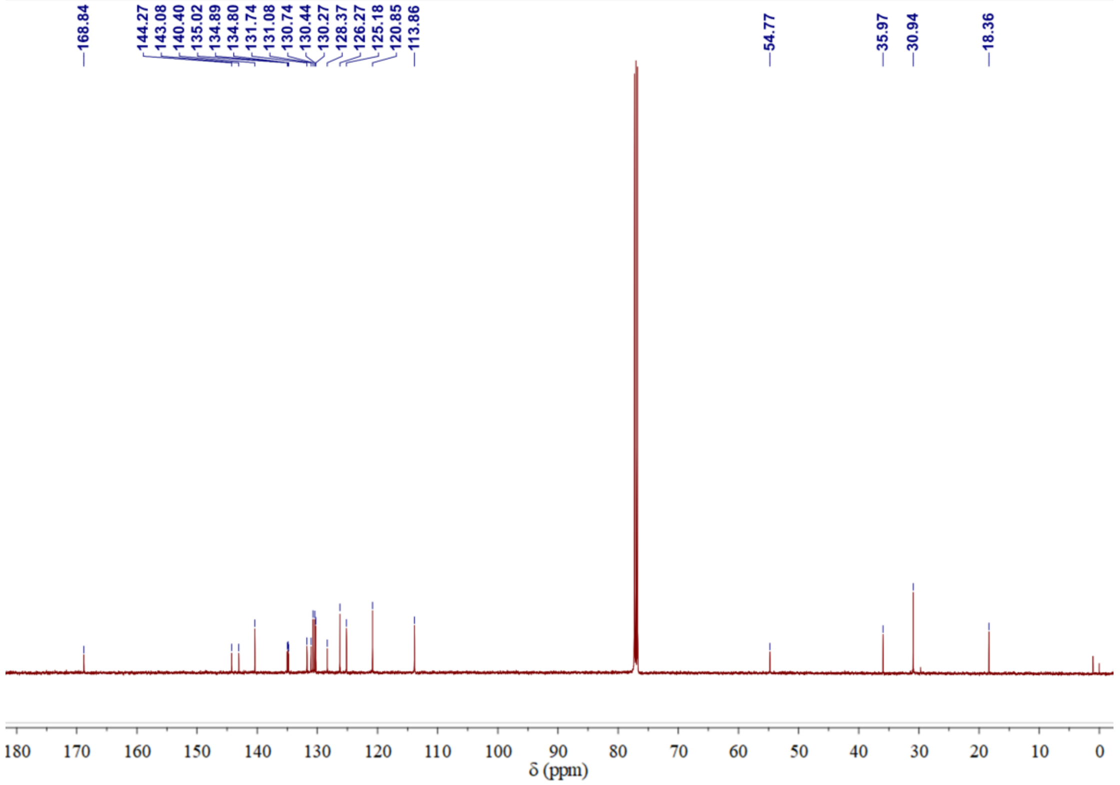

Figure S6. ^{13}C-NMR spectrum of **TB-DMAC** (126 MHz, $CDCl_3$)

2. Thermogravimetric Analysis (TGA)

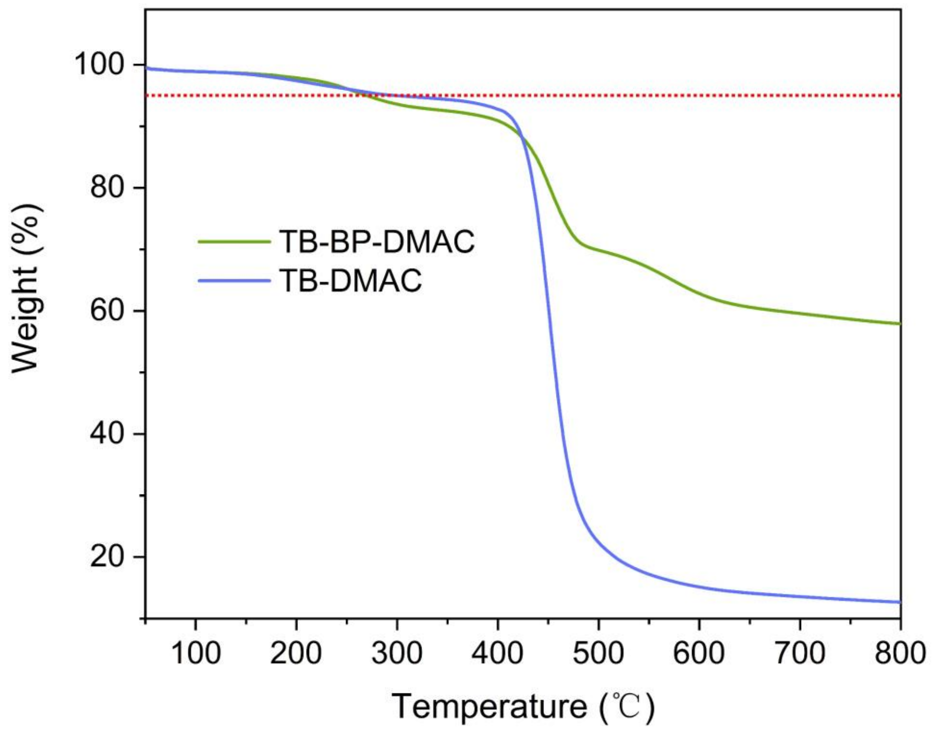

Figure S7. TGA curves of **TB-BP-DMAC** and **TB-DMAC** (The red dashed line marks 95% of the original sample weight).

3. Electrochemical Properties

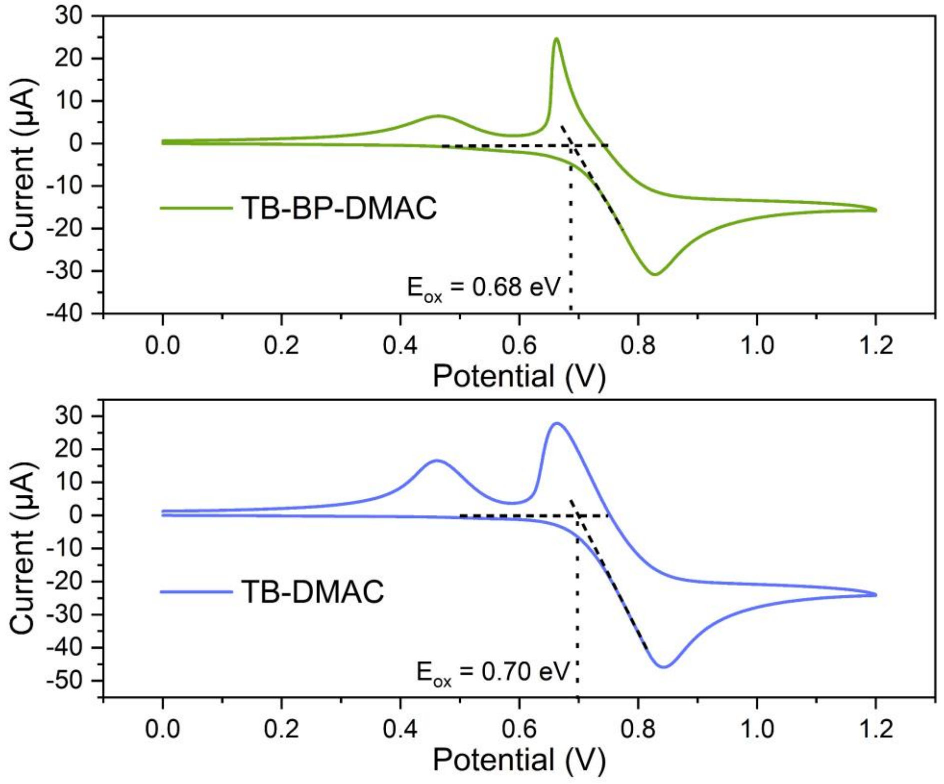

Figure S8. Cyclic Voltammograms of **TB-BP-DMAC** and **TB-DMAC**. Dashed vertical lines and intersections of tangent lines indicate the E_{ox} values.

The HOMO and LUMO energy levels were estimated from the cyclic voltammetry and optical bandgaps (E_g) determined from the onset of the absorption band (λ_{onset}).

$$E_{HOMO} = - [E_{ox} - E_{1/2(Fc/Fc+)} + 4.8] \text{ eV};$$

$$E_g = 1241/ \lambda_{onset};$$

$$E_{LUMO} = E_{HOMO} + E_g$$

Based on the above formulas, HOMO/LUMO energy levels of **TB-BP-DMAC** and **TB-DMAC** were calculated to be -5.26/-2.40 eV and -5.28/-2.08 eV, respectively.

Table S1. Summary of CV data and energy levels

Compound	E_{ox}[a] [eV]	$E_{1/2(Fc/Fc+)}$[b] [eV]	E_g[c] [eV]	E_{HOMO}[d] [eV]	E_{LUMO}[e] [eV]
**TB-BP-DMAC**	0.68	0.23	2.86	-5.26	-2.40
**TB-DMAC**	0.70	0.22	3.19	-5.28	-2.08

a) The oxidation potentials (E_{OX}) were acquired from the onset of first oxidation potentials in cyclic voltammograms (see Figure S8); b) ferrocenium/ferrocene couple was used as an internal standard; c) calculated from the absorption edge λ_{onset}; d) calculated from CV data; e) calculated from E_g and E_{HOMO}.

4. Theoretical Calculations

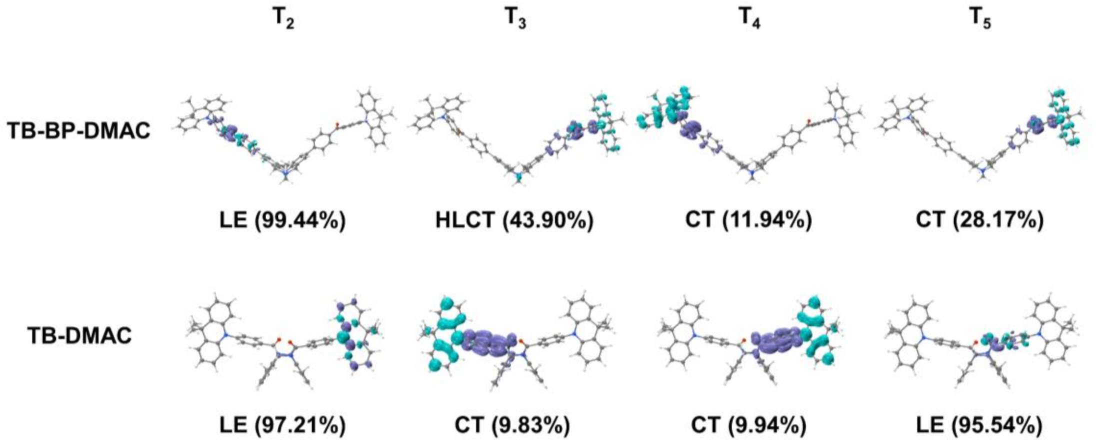

Figure S9. Electron-density distribution of the excited states (the purple area denotes an increase in charge density, while the blue area denotes a decrease in charge density) and character.

Table S2. Calculated spin-orbit coupling (SOC) constants.

SOC	S_1-T_1/cm^{-1}	S_1-T_2/cm^{-1}	S_1-T_3/cm^{-1}	S_1-T_4/cm^{-1}	S_1-T_5/cm^{-1}
**TB-BP-DMAC**	0.45	0.01	0	0	0.59
**TB-DMAC**	0.86	0.05	0	0	0.51

5. Photophysical Properties

5.1 Analysis of rate constants

Rate constants of the investigated compounds in 20 wt% PPF films at room temperature are determined from the measured quantum yields and lifetimes of the prompt fluorescence (PF) and delayed fluorescence (DF) components according to equations S1-S6 [1,2].

$$k_{PF} = \frac{1}{\tau_{PF}} \quad \text{Equation S1}$$

$$k_{DF} = \frac{1}{\tau_{DF}} \quad \text{Equation S2}$$

$$k_{RISC} = \frac{k_{PF}+k_{DF}}{2} - \sqrt{\left(\frac{k_{PF}+k_{DF}}{2}\right)^2 - k_{PF}k_{DF}(1+\frac{\Phi_{DF}}{\Phi_{PF}})}$$ Equation S3

$$k_{ISC} = \frac{k_{PF}k_{DF}}{k_{RISC}}\frac{\Phi_{DF}}{\Phi_{PF}}$$ Equation S4

$$k_r^S \approx \frac{k_{PF}k_{DF}}{k_{RISC}}\Phi_{PL}$$ Equation S5

$$k_{nr}^S \approx \frac{k_{PF}k_{DF}}{k_{RISC}}(1-\Phi_{PL})$$ Equation S6

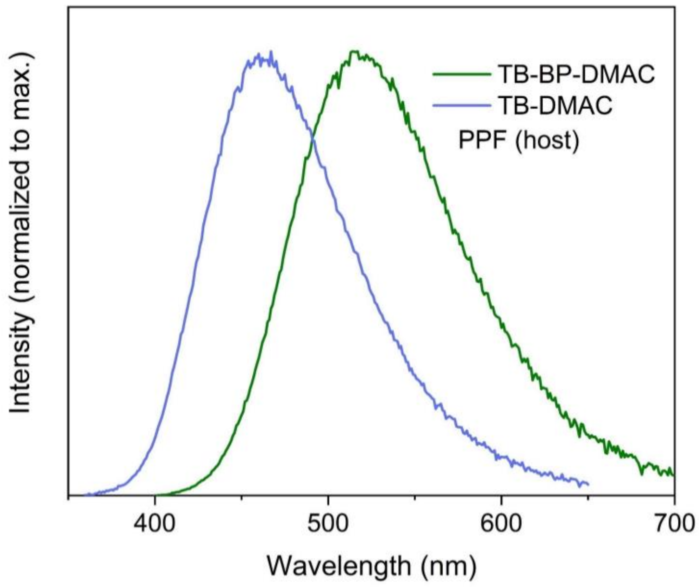

Figure S10. PL spectra (20 wt% -doped PPF films) measured at room temperature.

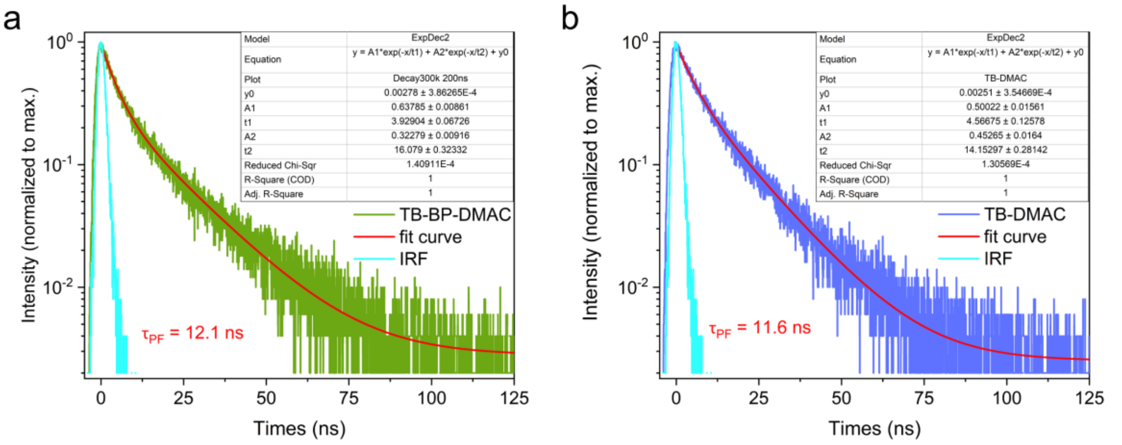

Figure S11. Transient PL decay curves (0-125 ns time-range, prompt decay) of (a) **TB-BP-DMAC** and (b) **TB-DMAC** in 20 wt%-doped PPF film at room temperature. Inset shows the fitting function along with the fitting results for the lifetimes of the prompt component, as well as the instrumental response function (IRF).

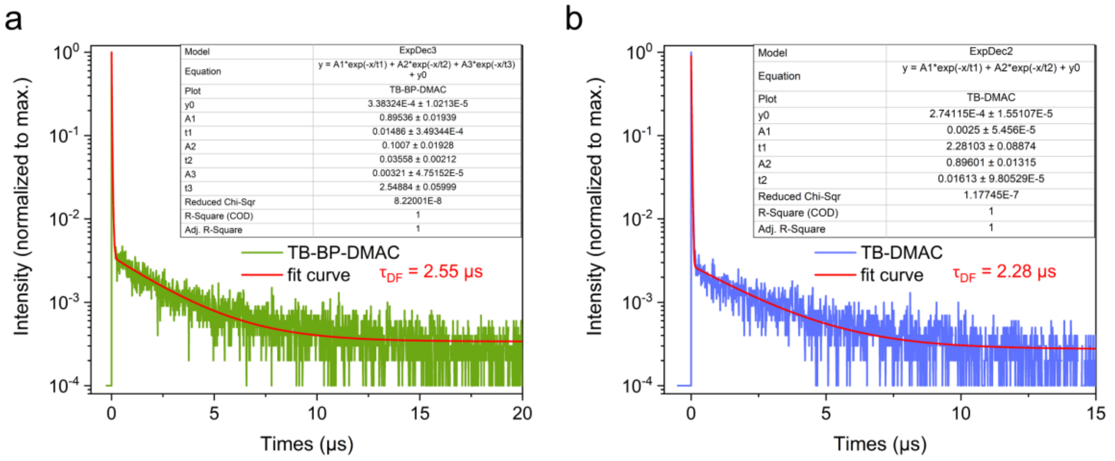

Figure S12. Transient PL decay curves of (a) **TB-BP-DMAC** and (b) **TB-DMAC** in 20 wt%-doped PPF film at room temperature. Inset shows the fitting function and fitting results of delayed component lifetimes.

Table S3. Fitting results of the transient PL decay curves of prompt fluorescence

Emitter	τ_1 [a] (ns)	Rel_1 [b] (%)	τ_2 [a] (ns)	Rel_2 [b] (%)	τ_{PF} [c] (ns)
**TB-BP-DMAC**	3.93	32.6	16.1	67.4	12.1
**TB-DMAC**	4.57	26.3	14.2	73.7	11.6

a) Lifetimes obtained by fitting the PL decay curves using 2-exponetial model function (Equation S7); b) The proportion of respective components, as a percentage; c) Average prompt fluorescence lifetime.

2-exponetial model function:

$$y = A_1 * exp\left(-\frac{x}{\tau_1}\right) + A_2 * exp\left(-\frac{x}{\tau_2}\right) + y_0 \qquad \text{Equation S7}$$

where, A_1 and A_2 are pre-exponential factors.

$$\tau_{PF} = (A_1 * \tau_1\text{^}2 + A_2 * t_2\text{^}2)/(A_1 * \tau_1 + A_2 * t_2) \qquad \text{Equation S8}$$

Table S4. Fitting results of the transient PL decay curves of delayed fluorescence

Emitter	τ_1 [a] (μs)	Rel_1 [b] (%)	τ_2 [a] (μs)	Rel_2 [b] (%)	τ_2 [a] (μs)	Rel_2 [b] (%)	τ_{DF} [c] (μs)	Rel_{DF} [d] (%)
**TB-BP-DMAC**	0.015	53.1	0.036	14.3	2.55	32.6	2.55	32.6
**TB-DMAC**	2.28	28.3	0.016	71.7	-	-	2.28	28.3

a) Lifetimes obtained by fitting the PL decay curves using 2/3-exponetial model function (Equation S7 and Equation S9); b) The proportion of respective components, as a percentage; c) Average delayed fluorescence lifetime; d) The proportion of delayed fluorescence, as a percentage.

3-exponetial model function:

$$y = A_1 * exp\left(-\frac{x}{\tau_1}\right) + A_2 * exp\left(-\frac{x}{\tau_2}\right) + A_3 * exp\left(-\frac{x}{\tau_3}\right) + y_0 \qquad \text{Equation S9}$$

where, A_1, A_2 and A_3 are pre-exponential factors.

Transient PL decay curves of the doped film samples at 300 K can be well fitted using 2/3-exponential model function (Equation S7 and Equation S9, also see **Figure S11 and Figure 12**). The proportion of each component were using:

$$Rel_1 = \frac{A_1*\tau_1}{A_1*\tau_1+A_2*\tau_2+A_3*\tau_3} \cdot 100\% \qquad \text{Equation S10}$$

$$Rel_2 = \frac{A_2*\tau_2}{A_1*\tau_1+A_2*\tau_2+A_3*\tau_3} \cdot 100\% \qquad \text{Equation S11}$$

$$Rel_2 = \frac{A_3*\tau_3}{A_1*\tau_1+A_2*\tau_2+A_3*\tau_3} \cdot 100\% \qquad \text{Equation S12}$$

The prompt fluorescence lifetimes of **TB-BP-DMAC** and **TB-DMAC** are denoted as τ_{PF} in **Table S3**, while the delayed fluorescence lifetimes of **TB-BP-DMAC** and **TB-DMAC** are represented as τ_{DF} in **Table S4**. PF and DF efficiencies (Φ_{PF} and Φ_{DF}) could be calculated to be 0.212 and 0.103 for **TB-BP-DMAC**, and 0.361 and 0.143 for **TB-DMAC**, respectively, by using Equation S13 and Equation S14.

$$\Phi_{DF} = (\mathrm{Rel}_{DF}) \times \Phi_{PL} \qquad \text{Equation S13}$$

$$\Phi_{PF} = (1 - \mathrm{Rel}_{DF}) \times \Phi_{PL} \qquad \text{Equation S14}$$

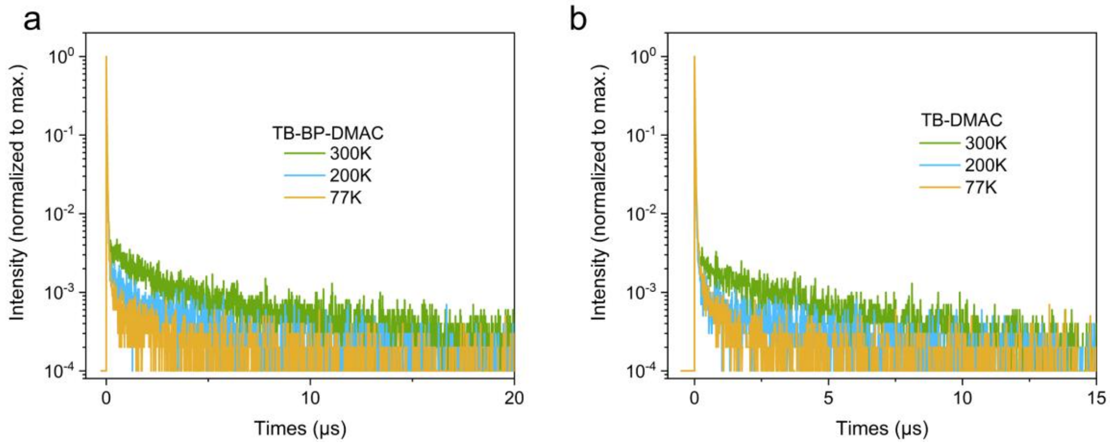

Figure S13. Transient PL decay curves of (a) **TB-BP-DMAC** and (b) **TB-DMAC** in 20 wt%-doped PPF film at different temperatures.

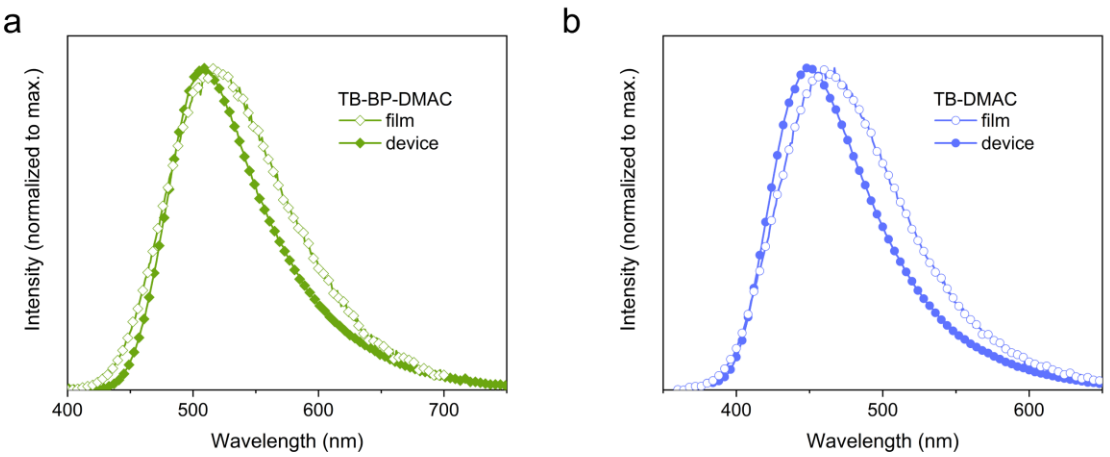

Figure S14. The comparison of EL (20 wt%-doped OLEDs) and PL spectra (20 wt%-doped films) of **TB-BP-DMAC** and **TB-DAMC**. The EL spectra were recorded at 8 V. The PL spectra were obtained using 300 nm excitation. All spectra were obtained at room temperature.

Table S5. Summary of ΔE_{ST} values for selected deep-blue and green TADF emitters.

	TADF emitter	λ_{EL} [nm]	ΔE_{ST} [eV]	ref.
deep-blue	PhCz-TOSBA	456	0.23	[3]
	TPA-TOSBA	456	0.36	
	BuCz-TRZ	459	0.29	[4]
	PhBuCz-TRZ	458	0.27	
	PyBuCz-TRZ	455	0.30	
	BuCz-MeTRZ	462	0.25	
	PyBuCz-MeTRZ	452	0.24	
	CNBuCz-TRZ	434	0.43	
	CzoB	455	0.15	[5]
	tBuO-SOpAD	445	0.07	[6]
	tBuO-SOmAD	435	0.17	
green	Cz-mCNTrz	492	0.03	[7]
	DCz-mCNTrz	504	0.02	
	TCz-mCNTrz	512	0.01	
	mCP-BP-DMAC	504	0.02	[8]
	DCB-BP-DMAC	498	0.04	
	**TB-BP-DMAC**	508	0.13	this work
	**TB-DMAC**	449	0.16	

6. Device Fabrication and Characterization

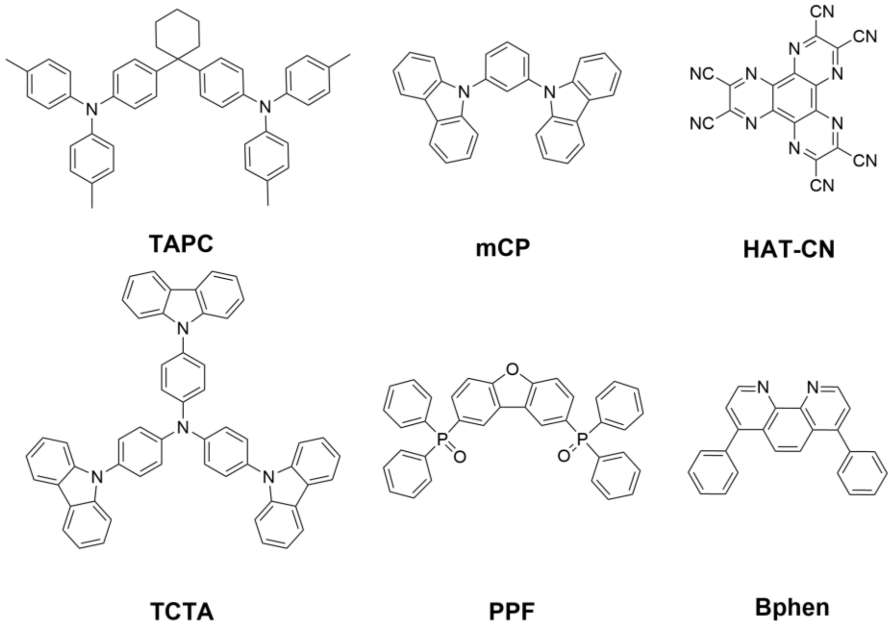

Figure S15. Molecular structures of the functional materials used in the OLEDs.

7. Reference

1. Wada, Y.; Nakagawa, H.; Matsumoto, S.; Wakisaka, Y.; Kaji, H. Organic light emitters exhibiting very fast reverse intersystem crossing. *Nat. Photonics* **2020**, *14*, 643-649, DOI: 10.1038/s41566-020-0667-0.
2. Chen, X-L.; Tao, X-D.; Wei, Z.; Meng, L.; Lin, F-L.; Zhang, D-H.; Jing, Y-Y.; Lu, C-Z. Thermally Activated Delayed Fluorescence Amorphous Molecular Materials for High-Performance Organic Light-Emitting Diodes. *ACS Appl. Mater. Interfaces* **2021**, *13*, 46909-46918, DOI: 10.1021/acsami.1c12188.
3. Gao, H.; Shen, S.; Qin, Y.; Liu, G.; Gao, T.; Dong, X.; Pang, Z.; Xie, X.; Wang, P.; Wang, Y. Ultrapure Blue Thermally Activated Delayed Fluorescence (TADF) Emitters Based on Rigid Sulfur/Oxygen-Bridged Triarylboron Acceptor: MR TADF and D-A TADF. *J. Phys. Chem. Lett.* **2022**, *13*, 7561-7567, DOI: 10.1021/acs.jpclett.2c01745.
4. Liu, B.; Li, J.; Liu, D.; Mei, Y.; Lan, Y.; Song, K.; Li, Y.; Wang, J. Electron-withdrawing bulky group substituted carbazoles for blue TADF emitters: Simultaneous improvement of blue color purity and RISC rate constants. *Dyes Pigm.* **2022**, *203*, 110329, DOI: 10.1016/j.dyepig.2022.110329.
5. Lee, Y. H.; Park, S.; Oh, J.; Shin, J. W.; Jung, J.; Yoo, S.; Lee, M. H. Rigidity-Induced Delayed Fluorescence by Ortho Donor-Appended Triarylboron Compounds: Record-High Efficiency in Pure Blue Fluorescent Organic Light-Emitting Diodes. *ACS Appl. Mater. Interfaces* **2017**, *9*, 24035-24042, DOI: 10.1021/acsami.7b05615.
6. Xia, Y.; Li, J.; Chen, X.; Li, A.; Guo, K.; Chen, F.; Zhao, B.; Chen, Z.; Wang, H. Molecular Engineering of Push-Pull Diphenylsulfone Derivatives towards Aggregation-Induced Narrowband Deep Blue Thermally Activated Delayed Fluorescence (TADF) Emitters. *Chemistry* **2022**, *28*, e202202434, DOI: 10.1002/chem.202202434.
7. Yoo, J-Y.; Choi, Y. J.; Kim, K. W.; Ha, T. H.; Lee, C. W. Improved device efficiency and lifetime of green thermally activated delayed fluorescence materials with multiple donors and cyano substitution. *Dyes Pigm.* **2023**, *214*, 111200, DOI: 10.1016/j.dyepig.2023.111200.
8. Wu, X.; Zeng, J.; Peng, X.; Liu, H.; Tang, B. Z.; Zhao, Z. Robust sky-blue aggregation-induced delayed fluorescence materials for high-performance top-emitting OLEDs and single emissive layer white OLEDs. *Chem. Eng. J.* **2023**, *451*, 138919, DOI: 10.1016/j.cej.2022.138919.
